# T2Bacteria and T2Resistance Assays in Critically Ill Patients with Sepsis or Septic Shock: A Descriptive Experience

**DOI:** 10.3390/antibiotics11121823

**Published:** 2022-12-15

**Authors:** Daniele Roberto Giacobbe, Francesca Crea, Paola Morici, Laura Magnasco, Vincenzo Di Pilato, Federica Briano, Edward Willison, Rachele Pincino, Silvia Dettori, Stefania Tutino, Simone Esposito, Erika Coppo, Chiara Dentone, Federica Portunato, Malgorzata Mikulska, Chiara Robba, Antonio Vena, Denise Battaglini, Iole Brunetti, Lorenzo Ball, Paolo Pelosi, Anna Marchese, Matteo Bassetti

**Affiliations:** 1 Department of Health Sciences (DISSAL), University of Genoa, 16132 Genoa, Italy; 2 Infectious Diseases Unit, San Martino Policlinico Hospital-IRCCS for Oncology and Neurosciences, 16132 Genoa, Italy; 3 Microbiology Unit, San Martino Policlinico Hospital-IRCCS for Oncology and Neurosciences, 16132 Genoa, Italy; 4 Department of Surgical Sciences and Integrated Diagnostics (DISC), University of Genoa, 16132 Genoa, Italy; 5 Ospedale San Paolo—ASL 2 Savonese, 17100 Savona, Italy; 6 Ospedale di Sanremo—ASL 1 Imperiese, 18038 Sanremo, Italy; 7 Anesthesia and Intensive Care, San Martino Policlinico Hospital-IRCCS for Oncology and Neurosciences, 16132 Genoa, Italy

**Keywords:** T2, rapid tests, diagnosis, sepsis, septic shock, BSI, antimicrobial resistance

## Abstract

The use of rapid molecular tests may anticipate the identification of causative agents and resistance determinants in the blood of critically ill patients with sepsis. From April to December 2021, all intensive care unit patients with sepsis or septic shock who were tested with the T2Bacteria and T2Resistance assays were included in a retrospective, single center study. The primary descriptive endpoints were results of rapid molecular tests and concomitant blood cultures. Overall, 38 combinations of T2Bacteria and T2Resistance tests were performed. One or more causative agent(s) were identified by the T2Bacteria assay in 26% of episodes (10/38), whereas negative and invalid results were obtained in 66% (25/38) and 8% (3/38) of episodes, respectively. The same pathogen detected by the T2Bacteria test grew from blood cultures in 30% of cases (3/10). One or more determinant(s) of resistance were identified by the T2Resistance assay in 11% of episodes (4/38). Changes in therapy based on T2Bacteria and/or T2Resistance results occurred in 21% of episodes (8/38). In conclusion, T2Bacteria/T2Resistance results can influence early treatment decisions in critically ill patients with sepsis or septic shock in real-life practice. Large, controlled studies remain necessary to confirm a favorable impact on patients’ outcomes and antimicrobial stewardship interventions.

## 1. Introduction

Bloodstream infections (BSI) caused by multidrug-resistant (MDR) bacteria remain associated with high mortality [1,2,3]. The clinical picture of BSI may be dominated by the development of sepsis or septic shock, which usually triggers the collection of blood cultures (aiming at confirming the diagnosis of BSI, as well as identifying the causative agent(s) and their susceptibility profile) and the initiation of empirical antibiotic therapy while waiting for blood cultures results [4,5,6,7].

Since blood culture results (identification and susceptibility test) usually become available after 48–96 h from the onset of the infection [8,9,10], the use of rapid molecular tests able to anticipate the identification of causative agents and clinically relevant resistance determinants in the blood of patients with sepsis or septic shock could reduce both the time of appropriate therapy and the misuse of antibiotics (by allowing early escalation or de-escalation) [8,11,12]. The T2Bacteria and T2Resistance assays are rapid molecular tests performed on whole blood that exploit T2 Magnetic Resonance (T2MR^®^) technology (T2Biosystems, Lexington, MA, USA) [13,14,15,16]. This technology allows the detection of the agglomeration of superparamagnetic particles induced by the amplicons, thereby leading to the identification of different bacteria with the T2Bacteria assay (*Enterococcus faecium*, *Staphylococcus aureus*, *Klebsiella pneumoniae*, *Pseudomonas aeruginosa*, *Acinetobacter baumannii*, and *Escherichia coli*) and of different resistance determinants with the T2Resistance assay (bla_KPC_, bla_OXA-48_, bla_NDM_, bla_VIM_, bla_IMP_, bla_CTX-M-14/15_, bla_CMY_, bla_DHA_, vanA/B, mecA/C), within 3–5 h after the blood draw [13,17,18].

In the present single center experience, we describe the use of the T2Bacteria and T2Resistance assays in critically ill patients with sepsis or septic shock, as well as the influence of their results on treatment decisions.

## 2. Methods

### 2.1. Setting and Study Design

The present retrospective study was conducted in intensive care units (ICUs) of San Martino Policlinico Hospital-IRCCS for Oncology and Neurosciences, a 1200-bed teaching hospital in Northern Italy. At the time of the study, the hospital had five ICUs: (i) one for general ICU patients and neurosurgical patients, with 28 beds; (ii) one for coronavirus disease 2019 (COVID-19) and respiratory patients, with 10 beds; (iii) one for cardiovascular surgery patients, with 12 beds; (iv) one for major surgery and solid organ transplant patients, with 10 beds; (v) one in the emergency department, with 8 beds. From 1 April 2021 to 31 December 2021, all ICU patients with sepsis or septic shock who were tested with the T2Bacteria and the T2Resistance assays at the clinical onset of sepsis/septic shock were included in the study. Decisions about which patients to test were made by infectious diseases consultants according to local practice, based on personal judgment and balancing different factors (e.g., consultation requested at the time of sepsis/septic shock onset, lack of 24/7 laboratory service for T2 assays). Patients could be included more than once if they were tested during different sepsis/septic shock episodes. Blood cultures were also performed in all episodes at the onset of sepsis/septic shock, according to standard practice. Sepsis and septic shock were defined according to Sepsis-3 criteria [19]. The primary descriptive endpoints were results of T2Bacteria/T2Resistance assays and concomitant blood cultures. Secondary descriptive endpoints were changes of antibiotic therapy according to T2Bacteria/T2Resistance results (either positive or negative) and crude 30-day mortality after the onset of sepsis/septic shock.

### 2.2. Microbiological Evaluation

For each episode, two whole blood samples for the T2Bacteria Panel and T2Resistance panel assays were collected into 4-mL K2 EDTA Vacutainer blood collection tubes. T2 specimens were processed immediately upon their arrival at the laboratory by a fully automated T2Dx instrument. The T2Bacteria assay allows detection of the following bacteria: *Enterococcus faecium*; *Staphylococcus aureus*; *Klebsiella pneumoniae*; *Pseudomonas aeruginosa*; *Acinetobacter baumannii*; *Escherichia coli*. The T2Resistance assay allows detection of the following resistance determinants: bla_KPC_; bla_OXA-48_; bla_NDM_; bla_VIM_; bla_IMP_; bla_CTX-M-14/15_; bla_CMY_; bla_DHA_; vanA/B; mecA/C. Blood culturing was performed for 5–7 days in accordance with routine laboratory practice using the automated Bactec FX system (Becton-Dickinson, Franklin Lakes, NJ, USA). Positive blood cultures were subjected to Gram staining microscopy and solid-medium subcultures. The Vitek MS MALDI-TOF system (software v 4.0; bioMérieux, Marcy l’Etoile, France) was routinely used for identifying microorganisms isolated from blood culture, whereas the Vitek 2 AES system was used for susceptibility testing. The results of the susceptibility tests were interpreted according to the criteria of the European Committee on Antimicrobial Susceptibility Testing (EUCAST) (breakpoint tables for interpretation of minimum inhibitory concentrations [MIC] and zone diameters, version 11.0, 2021 http://www.eucast.org, accessed on 26 October 2022). The immunochromatographic rapid diagnostic test RESIST-4 O.K.N.V. RDT (Coris BioConcept; Gembloux, Belgium) was used to detect different carbapenemases in culture-grown Gram-negative bacteria.

### 2.3. Statistical Analysis

No a priori sample size calculation was performed for this preliminary, exploratory study. Descriptive endpoints are reported by number and percentages. Exact confidence intervals for percentages were calculated with the Blaker’s method [20]. Descriptive comparison of 30-day mortality was performed with the chi-square test between episodes in which antibiotic therapy was changed according to T2Bacteria and/or T2Resistance results and episodes in which antibiotic therapy was not changed following T2Bacteria and/or T2Resistance results.

## 3. Results

During the study period, 38 T2Bacteria and T2 Resistance paired tests were performed on whole blood from 33 ICU patients with sepsis or septic shock. Their median age was 62 years (interquartile range 47–71) and 55% were males (18/33). The most frequently suspected source of infection was the lower respiratory tract (13/38, 34%), followed by the abdomen (8/38, 21%), whereas endocarditis, mediastinitis, and the urinary tract were the suspected primary source of infection in one episode each. The source of infection was unknown/unclear in the remaining 37% of episodes (14/38). Additional details on the demographic and clinical characteristics of patients are available in Supplementary Table S1.

The results of T2Bacteria and T2Resistance tests, as well as their concordance with blood cultures results, are shown in Table 1. Overall, one or more causative agent(s) were identified by the T2Bacteria assay in 26% of episodes (10/38), whereas negative and invalid results were obtained in 66% (25/38) and 8% (3/38) of episodes, respectively. Excluding invalid results, the concordance rate between T2Bacteria and blood cultures results was 66% (23/35). One or more determinant(s) of resistance were identified by the T2Resistance assay in 11% of episodes (4/38), whereas negative and invalid results were obtained in 79% (30/38) and 11% (4/38) of episodes, respectively. The same pathogen detected by the T2Bacteria assay grew from concomitantly collected blood cultures in 30% of episodes (3/10), whereas no growth was observed from concomitant blood cultures in 86% (6/7) of the other episodes with positive T2Bacteria results (in the remaining 1/7 episode, *Candida auris* grew from blood cultures in presence of a T2Bacteria test positive for *Klebsiella pneumoniae*).

In episodes with negative T2Bacteria results, positive blood cultures were obtained in five cases (one for *Klebsiella oxytoca*, one for *Citrobacter freundii*, one for *Staphylococcus epidermidis*, and two for *Candida auris*). In one of the three episodes with invalid T2Bacteria results (33%), blood cultures turned out positive for *K. pneumoniae*, whereas they were negative in the two remaining cases. In one of the four episodes with positive T2Resistance results (25%), results were consistent with those obtained from the standard laboratory workflow (KPC-producing *K. pneumoniae*), whereas discordant results were observed in the other three episodes with positive T2Resistance results (one for bla_KPC_ and bla_CTX-M-14/15_, one for bla_CTX-M-14/15_, and one for vanA/B), since no growth was observed from blood cultures.

**Figure 1:**
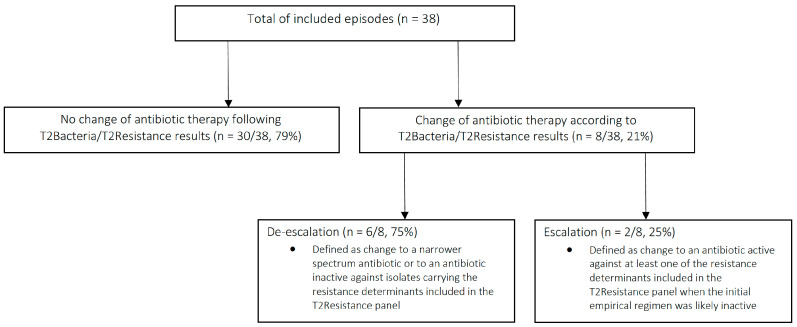
Changes in antibiotic therapy according to T2Bacteria/T2Resistance results before availability of blood cultures results.

As shown in Figure 1, changes of antibiotic therapy based on T2Bacteria and/or T2Resistance results occurred in 21% of cases (8/38). Overall, 30-day mortality after the onset of sepsis/septic shock was 38% (3/8) when antibiotic therapy was changed according to T2Bacteria and/or T2Resistance results, and 57% (17/30) when antibiotic therapy was not changed following T2Bacteria and/or T2Resistance results (*p* = 0.334).

## 4. Discussion

In this preliminary, real-life experience, the results of the T2Bacteria and T2Resistance assays led to early changes in antibiotic therapy in 21% of critically ill patients with sepsis or septic shock admitted to ICUs. 

In our opinion, two pragmatic clinical considerations stem from the present study. Firstly, T2Bacteria and T2Resistance assays can influence early treatment decision in real-life practice. The effectiveness of these laboratory tests should be tested in large, controlled studies with respect to their impact on clinically relevant endpoints. With regard to the T2Bacteria assay, Quirino and colleagues [21], in an underpowered clinical study, have previously reported a lower 21-day mortality in patients with suspected BSI who underwent T2Bacteria and T2Candida testing in comparison with patients with suspected BSI who did not underwent such rapid tests (22% vs. 44%, respectively). Secondly, in 79% of the episodes in our study, the antibiotic regimen was not changed despite availability of T2Bacteria/T2Resistance results. In our opinion, this does not reflect low confidence in rapid tests results. This was more likely the consequence of the complex clinical reasoning guiding early treatment decisions based on rapid tests results. Indeed, clinicians evaluating the results of molecular rapid tests also need to consider organisms (e.g., *Citrobacter* spp. or *Klebsiella oxytoca*, which were detected by blood cultures in some episodes in our study) and resistance determinants (e.g., mechanisms of carbapenem resistance in *Pseudomonas aeruginosa* other than β-lactamases) that are not covered by the T2 panels, and thus cannot be detected before blood cultures’ results. From this standpoint, a decision of continuing the initial regimen could still reflect appropriateness (e.g., no escalation deemed necessary in the case of negative T2Bacteria and T2Resistance results) and potential usefulness of the T2 assays. The possibility of negative T2 results in the presence of a non-bacteremic sepsis/septic shock should also be weighed in balance when making early treatment decisions in patients already colonized by MDR bacteria. In our opinion, there is a need for training and expertise in interpretating T2Bacteria and T2Resistance results at the bedside of critically ill patients with sepsis or septic shock. Indeed, it cannot be excluded a priori that a treatment change decision taken without balancing all factors discussed above could be harmful. For this reason, in our real-life experience, T2-guided decisions were always made by infectious diseases specialists. In this context, diagnostic stewardship is necessary to optimize the benefits of syndromic testing and to ensure a correct interpretation of the results in the context of the patient. In our analysis, we compared crude 30-day mortality between episodes in which treatment was changed based on T2Bacteria/T2Resistance results and episodes in which treatment remained unchanged, to confirm that no increase in mortality occurred after changes in treatment based on T2 results.

Overall, the global impact of T2Bacteria/T2Resistance on clinical outcomes at population level needs to be assessed in controlled studies. Nonetheless, we believe that our preliminary results can be considered promising regarding the potential for T2-guided early escalation/de-escalation in critically ill patients with sepsis/septic shock, for improving either clinically relevant outcomes or antimicrobial stewardship interventions (e.g., rapid narrowing of initial broad-spectrum antibiotic therapy and anticipation of appropriate antibiotic therapy). This hypothesis is in line with the findings of a recent systematic review and meta-analysis summarizing the favorable impact on resources utilization of T2Candida and T2Bacteria assays [14].

The main limitations of the present study are the small sample size of our study population, the lack of a control group, and the unavailability of the T2Candida assay in our hospital at the time of the study (which could have changed the eventual number of treatment changes based on T2 results). Chance related to the small sample could also explain our 66% concordance rate between T2Bacteria and blood cultures results, which was lower than in other studies [16,18], although it is of note that most discordant results were negative blood cultures and positive T2Bacteria results. In this latter case, the existence of a true episode of systemic infection cannot be ruled out. Finally, the retrospective nature of the analysis precluded the collection of more detailed information on the precise reasons leading to treatment decisions based on T2Bacteria/T2Resistance results.

## 5. Conclusions

T2Bacteria/T2Resistance results can influence early treatment decisions in critically ill patients with sepsis or septic shock in real-life practice. Large, controlled studies are warranted to confirm a favorable impact on clinically relevant patients’ outcomes and antimicrobial stewardship interventions.

## Figures and Tables

**Table 1:** Distribution of results of T2Bacteria/T2Resistance panels and conventional blood cultures/antimicrobial susceptibility testing.

Results Category *n* (%, 95% CI) **	T2Bacteria Results *	T2Resistance Results *	Conventional Methods Results *	Isolation of the Same Bacteria Detected by T2 in Other Clinical Samples
Positive T2Bacteria *n* = 10/38 (26%, 14–42)	*K. pneumoniae* (*n* = 2) *P. aeruginosa n* = 2) *A. baumannii* *E. faecium* *K. pneumoniae* *K. pneumoniae* *P. aeruginosa* *P. aeruginosa* and *K. pneumoniae*	Negative (*n* =2) Negative (*n* = 2) Negative *VanA/B* *bla*_KPC_ *bla*_KPC_ and *bla*_CTX-M-14/15_ *bla*_CTX-M-14/15_ Negative	Negative (*n* = 2) Carbapenem-susceptible *P. aeruginosa* (*n* = 2) Negative Negative Carbapenem-resistant, KPC-producing *K. pneumoniae* *C. auris* Negative Negative	- - - Peritoneal fluid Respiratory Urine, respiratory Respiratory -
Negative T2Bacteria *n* = 25/38 (66%, 49–80)	Negative (*n* = 16) Negative (*n* = 4) Negative (*n* = 2) Negative Negative Negative	Negative (*n* = 16) Invalid (*n* = 4) Negative (*n* = 2) Negative Negative Negative	Negative (*n* = 16) Negative (*n* = 4) *C. auris* (*n* = 2) Carbapenem-susceptible *C. freundii* Carbapenem-susceptible *K. oxytoca* Methicillin-resistant *S. epidermidis* ***	
Invalid T2Bacteria *n* = 3/38 (8%, 2–20)	Invalid (*n* = 2) Invalid	Negative (*n* = 2) Negative	Negative (*n* = 2) Carbapenem-susceptible *K. pneumoniae* and *C. parapsilosis*	

* One line per episode unless otherwise specified. ** Exact confidence intervals were calculated with the Blaker’s method. *** Two different positive blood cultures for methicillin-resistant *Staphylococcus epidermidis*.

## Data Availability

The data presented in this study are available on reasonable request from the corresponding author.

## Supplementary Materials

The following supporting information can be downloaded at: https://www.mdpi.com/article/10.3390/antibiotics11121823/s1, Supplementary Table S1: Demographic and clinical characteristics of 33 critically ill patients tested with T2Bacteria and T2Resistance assays.
