# Supplementary material for: T2Bacteria and T2Resistance Assays in Critically Ill Patients with Sepsis or Septic Shock: A Descriptive Experience
Source: Antibiotics (Basel). 2022 Dec 15;11(12):1823. doi: 10.3390/antibiotics11121823 (PMC9774778; doi:10.3390/antibiotics11121823)
Supplement: Supplementary file 1 [file antibiotics-11-01823-s001.zip › antibiotics-2026317-supplementary.pdf]

Supplementary table S1. Demographic and clinical characteristics of 33 critically ill patients tested with T2Bacteria and T2Resistance assays

Variable	Total of patients [a] 33 (100)
**Demographic variables**	
Age in years, median (IQR)	62 (47-71)
Male gender	18 (55)
**Comorbidities**	
COVID-19	17 (52)
Hematological malignancy	2 (6)
Solid neoplasm	2 (6)
**Variables at time of T2 testing [b]**	
C-reactive protein in mg/L, median (IQR)	128.5 (57.4-166)
Procalcitonin in ng/mL, median (IQR)	1.11 (0.42-9.26)
Neutropenia (ANC < 500 cell/mm^3)	1 (3)
Septic shock	21 (55)
Antibiotic therapy	6 (16)

ANC, absolute neutrophil count; COVID-19, coronavirus disease 2019; IQR, interquartile range.
[a] Results are presented as No. of patients (%) unless otherwise indicated.
[b] Calculated for a denominator of 38 episodes occurring in 33 patients
[c] Already initiated from at least 24 hours at the time of T2 testing
